# Supplementary material for: The extracellular gate shapes the energy profile of an ABC exporter
Source: Nat Commun. 2019 May 21;10:2260. doi: 10.1038/s41467-019-09892-6 (PMC6529423; doi:10.1038/s41467-019-09892-6)
Supplement: Supplementary file 1 — Supplementary Information [file 41467_2019_9892_MOESM1_ESM.pdf]

Supplementary Information

The extracellular gate shapes the energy profile of an ABC exporter

Hutter et al.

Supplementary Table 1: Data collection and refinement statistics.

**PDB ID**	**6QUZ**	**6QV0**	**6QV1**	**6QV2**
	EtoA	2xDtoA/EtoA	2xDtoA/EtoA	2xDtoA/EtoA
	ATPγS-Mg	ATP-Mg	ATPγS-Mg	ATPγS-Mg
	Sb_TM#35	Sb_TM#35	Nb_TM#1	Nb_TM#2
**Data collection**				
Space group	$P2_1$	$P2_1$	$P2_1$	P1
Cell dimensions				
a, *b*, *c* (Å)	165.34, 77.11, 207.01	166.28, 77.29, 207.18	90.75, 199.26, 113.16	88.60, 113.07, 126.89
α, β, γ (°)	90.00, 112.49, 90.00	90.00, 112.55, 90.00	90.00, 91.26, 90.00	83.18, 73.00, 67.37
Resolution (Å)	49.14-3.20 (3.29-3.20)*	49.19-3.11 (3.19-3.11)	48.48-3.48 (3.57-3.48)	48.06-4.22 (4.32-4.22)
R_{meas}	0.26 (1.00) *[0.34 (3.97)]*#	0.20 (1.02) *[0.34 (6.62)]*	0.06 (0.85) *[0.09 (4.23)]*	0.06 (0.41) *[0.11 (3.11)]*
$I / \sigma I$	8.91 (3.03) *[7.30 (0.75)]*	9.25 (2.28) *[6.01 (0.41)]*	16.3 (3.26) *[9.77 (0.63)]*	16.1 (3.25) *[8.89 (0.52)]*
Completeness (%)	79.6 (7.2) *[99.8 (99.7)]*	62.6 (3.2) *[95.5 (97.6)]*	58.2 (2.9) *[98.8 (99.4)]*	57.4 (2.1) *[98.8 (98.7)]*
Redundancy	13.6 (14.3) *[13.7 (14.1)]*	6.7 (6.6) *[6.8 (6.9)]*	7.1 (7.7) *[7.2 (7.4)]*	3.5 (3.6) *[3.5 (3.5)]*
$CC_{1/2}$ (%)	99.8 (85.0) *[99.8 (51.0)]*	99.8 (77.2) *[99.6 (22.3)]*	100 (73.9) *[100 (40.2)]*	100 (83.8) *[100 (43.0)]*
**Refinement**				
Resolution (Å)	48.84-3.21	49.19-3.12	34.64-3.48	32.59-4.23
No. reflections	63'764	55'122	29'833	17'961
R_{work} / R_{free}	24.3/26.3	24.4/26.4	26.7/30.3	30.6/33.0
No. atoms				
Protein	19'970	19'958	19'788	19'958
Ligand/ion	128	128	128	128
B-factors				
Protein	72.3	72.0	190.1	167.9
Ligand/ion	46.6	50.9	134.9	146.7
R.m.s. deviations				
Bond lengths (Å)	0.008	0.008	0.008	0.008
Bond angles (°)	0.95	0.94	0.95	0.90

*Values in parentheses are for highest-resolution shell.

Italic values in brackets are before ellipsoidal truncation.

Supplementary Table 2: Kinetic parameters derived from SPR measurements.

		Sb_TM#35		Nb_TM#1		Nb_TM#2	
		- ATP + Mg^{2+}	+ ATP + Mg^{2+}	- ATP + Mg^{2+}	+ ATP + Mg^{2+}	- ATP + Mg^{2+}	+ ATP + Mg^{2+}
**TM287/288**	k_{on} ($M^{-1}s^{-1}$)	x	$1.43*10^{4}$	x	$3.41*10^{5}$	$2.18*10^{6}$	$2.77*10^{6}$
	k_{off} (s^{-1})	x	$1.57*10^{-3}$	x	$6.28*10^{-2}$	$2.06*10^{-3}$	$2.12*10^{-3}$
	K_D (M)	x	$1.10*10^{-7}$	x	$1.84*10^{-7}$	$9.43*10^{-10}$	$7.65*10^{-10}$
**TM287/288 EtoQ**	k_{on} ($M^{-1}s^{-1}$)	x	$2.06*10^{4}$	x	$1.01*10^{6}$	$2.44*10^{6}$	$2.36*10^{6}$
	k_{off} (s^{-1})	x	$1.35*10^{-3}$	x	$5.04*10^{-3}$	$1.96*10^{-3}$	$2.19*10^{-3}$
	K_D (M)	x	$6.56*10^{-8}$	x	$4.98*10^{-9}$	$8.03*10^{-10}$	$9.28*10^{-10}$
**TM287/288 EtoA**	k_{on} ($M^{-1}s^{-1}$)	x	$1.92*10^{4}$	x	$9.01*10^{5}$	n.d.	n.d.
	k_{off} (s^{-1})	x	$1.26*10^{-3}$	x	$4.86*10^{-3}$	n.d.	n.d.
	K_D (M)	x	$6.57*10^{-8}$	x	$5.40*10^{-9}$	n.d.	n.d.
**TM287/288 2xDtoA**	k_{on} ($M^{-1}s^{-1}$)	x	$1.84*10^{5}$	x	$4.27*10^{5}$	n.d.	n.d.
	k_{off} (s^{-1})	x	$2.55*10^{-3}$	x	$1.57*10^{-2}$	n.d.	n.d.
	K_D (M)	x	$1.38*10^{-8}$	x	$3.68*10^{-8}$	n.d.	n.d.
**TM287/288 EtoA 2xDtoA**	k_{on} ($M^{-1}s^{-1}$)	x	$2.82*10^{5}$	x	$8.92*10^{5}$	$2.40*10^{6}$	$2.44*10^{6}$
	k_{off} (s^{-1})	x	$2.36*10^{-3}$	x	$4.40*10^{-3}$	$1.61*10^{-3}$	$2.03*10^{-3}$
	K_D (M)	x	$8.34*10^{-9}$	x	$4.94*10^{-9}$	$6.73*10^{-10}$	$8.29*10^{-10}$

x: no SPR signal, n.d.: not determined

Supplementary Table 3:
ATPase activities of spin-labeled TM287/288 determined using 500 µM ATP at 25°C.

	ATPase activity (nmol Pi min^{-1} mg $protein^{-1}$)	Inhibited ATPase activity + 1.2 µM Sb_TM#35 (nmol Pi min^{-1} mg $protein^{-1}$)
wildtype	97.6 ± 6.7	21.7 ± 1.3
$54^{TM287}/290^{TM288}$	59.4 ± 3.7	22.1 ± 2.0
$54^{TM287}/271^{TM287}$	44.5 ± 3.6	14.1 ± 1.7
54^{TM287}	78.3 ± 4.4	23.7 ± 2.7
271^{TM287}	48.2 ± 3.0	21.3 ± 2.1

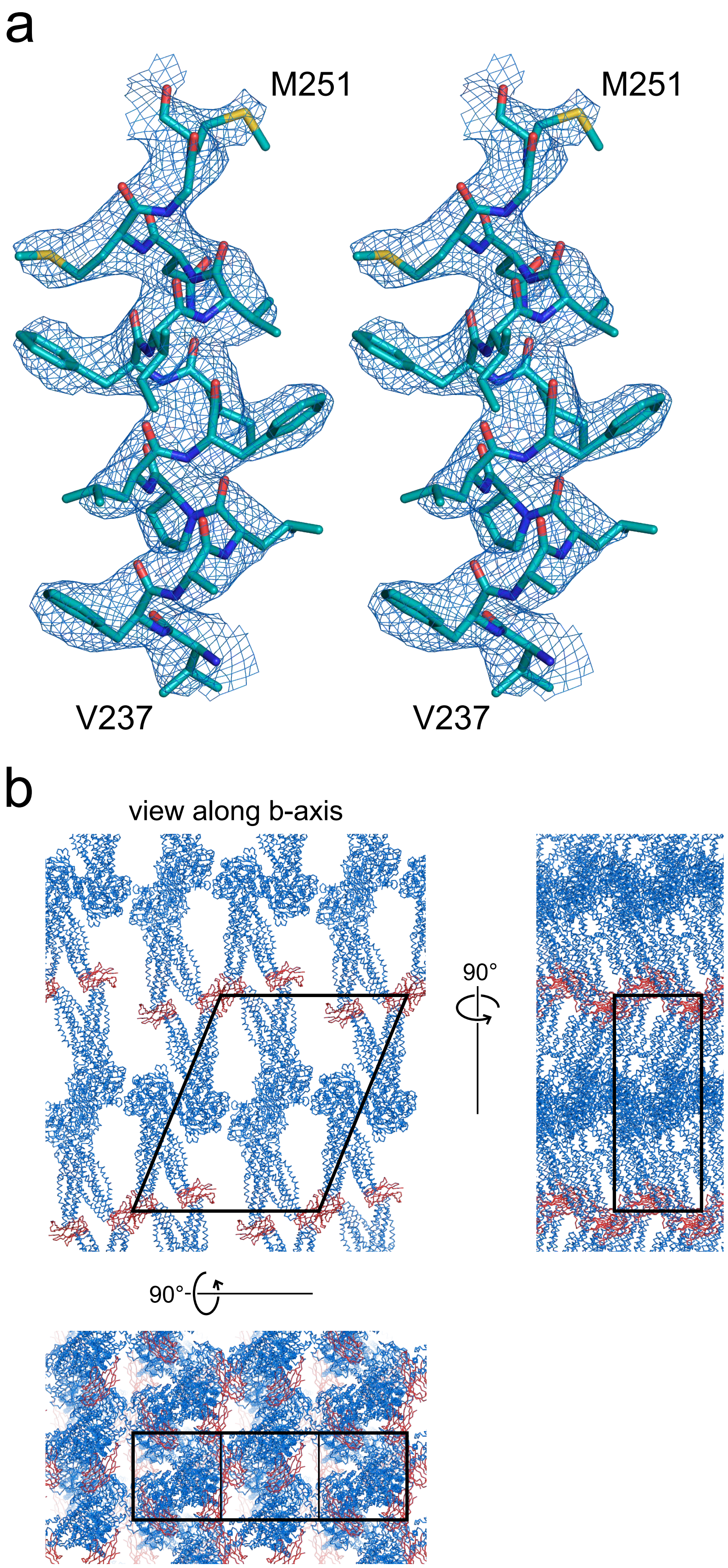

Supplementary Figure 1: Electron density and crystal packing. (**a**) Stereo view of a portion (6QUZ, chain A, residues V237 - M251) of the 2Fo-Fc electron density map contoured at 1.3 sigma (blue mesh). (**b**) Sybody Sb_TM#35 (red) is involved in crystal packing.

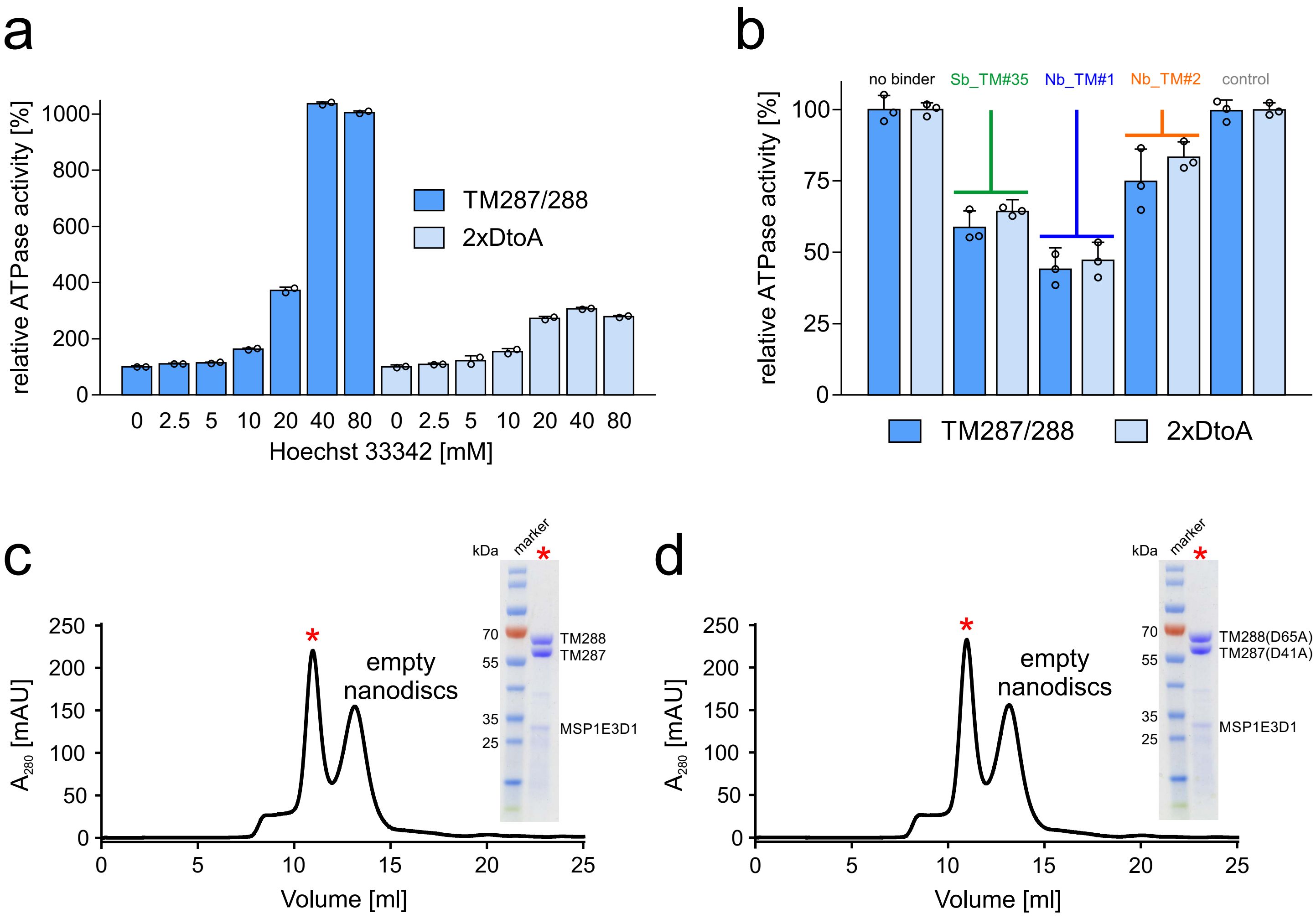

Supplementary Figure 2: Characterization of TM287/288 reconstituted in nanodiscs. TM287/288 wildtype and 2xDtoA mutant were reconstituted into nanodiscs as described in Methods. (**a**) ATPase activity in the absence (basal activity) or presence of increasing Hoechst 33342 concentrations. Data were normalized to the basal activity of wildtype transporter or 2xDtoA mutant, respectively. (**b**) Inhibition of Hoechst 33342 stimulated ATPase activities (50 µM) in the presence of the binders Sb_TM#35, Nb_TM#1 or Nb_TM#2 (10 µM). A non-randomized sybody (10 µM) served as control. Data were normalized to the ATPase activity measured in the absence of binders. The error bars are standard deviations of technical duplicates (a) or triplicates (b). (**c** and **d**) SEC profiles and SDS PAGE analysis of TM287/288 wildtype (c) and 2xDtoA (d) mutant reconstituted in nanodiscs. The peak marked with a red star contains the transporter reconstituted in nanodiscs and was analyzed by SDS PAGE and stained with Coomassie Brilliant Blue. Bands corresponding to TM287, TM288 and MSP1E3D1 are labelled.

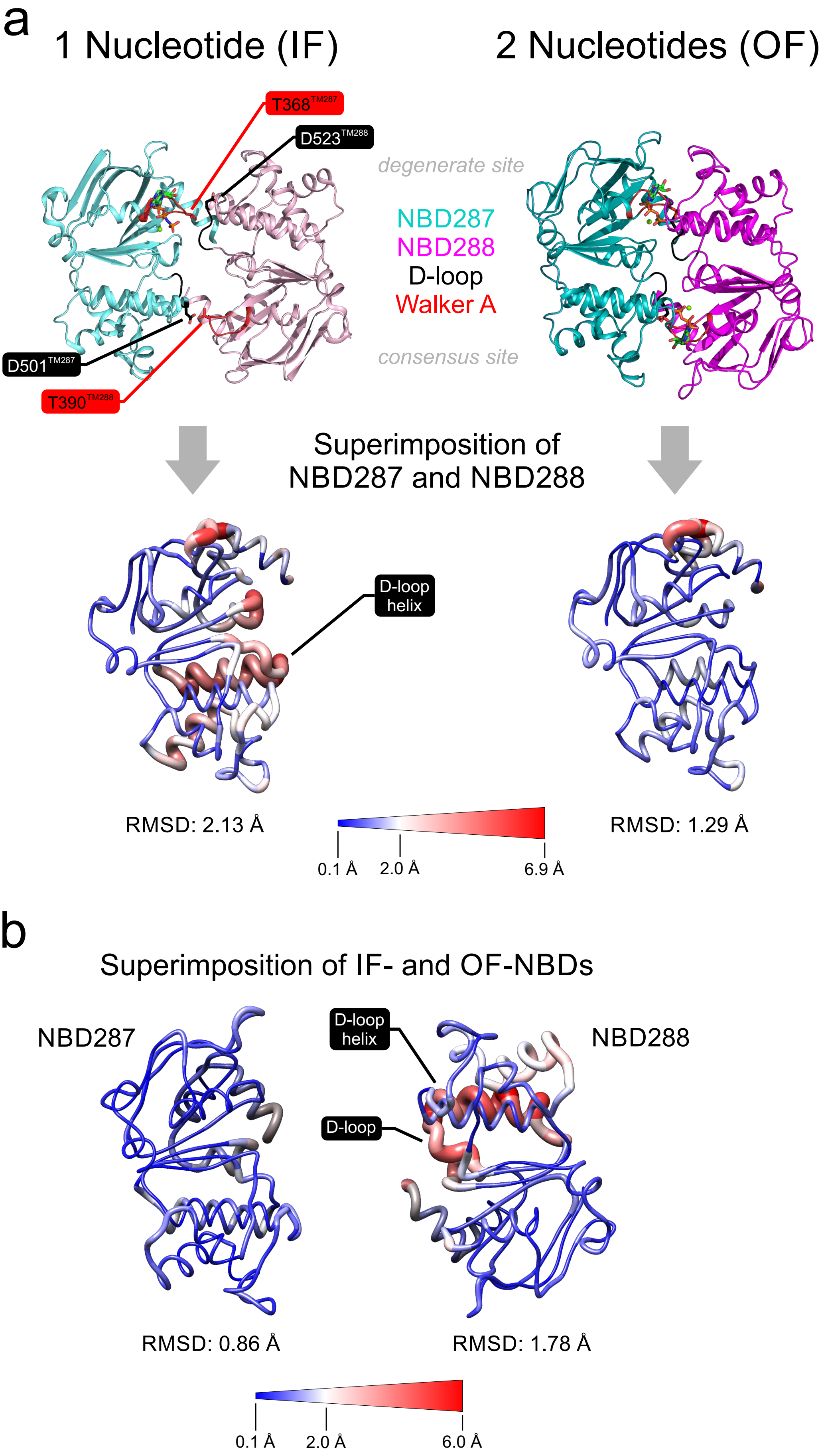

Supplementary Figure 3: Superimposition of NBDs. Superimpositions are shown as sausage representations in which greater thickness and red coloring correlate with larger structural deviations. (**a**) Superimposition of NBD287 with NBD288 of inward-facing (IF) TM287/288 with bound AMP-PNP-Mg (left, PDB ID: 4Q4A) or outward-facing (OF) with two bound ATPγS-Mg (right, PDB ID: 6QUZ). Strong asymmetries found at the D-loop and D-loop helix of the IF structure are resolved in the closed NBD dimer of the OF structure. (**b**) Structural changes within the same NBDs during IF-OF conversion. While NBD287 moves as a rigid body, large rearrangements occur at the D-loop and D-loop helix of NBD288.

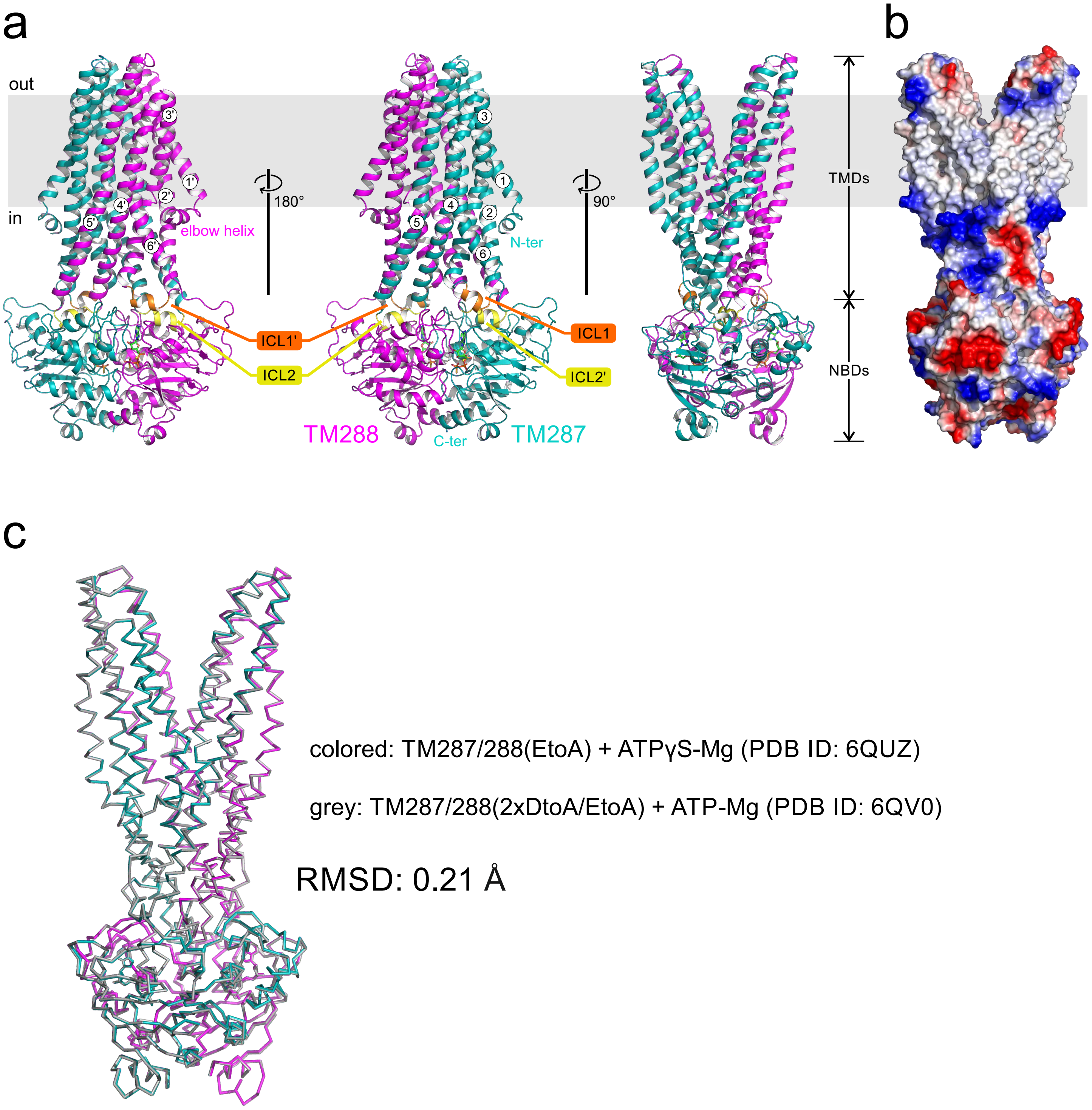

Supplementary Figure 4: Side view of outward-facing TM287/288. (**a**) Cartoon representation of TM287/288 (PDB ID: 6QUZ, sybody removed) is colored in teal (TM287) and magenta (TM288) seen from different angles. The grey rectangle shows the membrane region. The twelve transmembrane helices are numbered (1-6 for TM287 and 1'-6' for TM288). Intracellular loop 1 (ICL1) of TM287/288 features a kink that is not seen in ICL1' of TM288 and is not common in ABC exporter structures. (**b**) Surface representation of outward-facing TM287/288 (positive and negative charges in blue and red, respectively). Hydrophobic and amphiphilic substrates could exit the binding cavity while remaining partially embedded in the outer leaflet of the lipid bilayer. (**c**) Superimposition of outward-facing TM287/288 structures determined with Sb_TM#35 in the presence of ATPγS-Mg (colored) or ATP-Mg (grey).

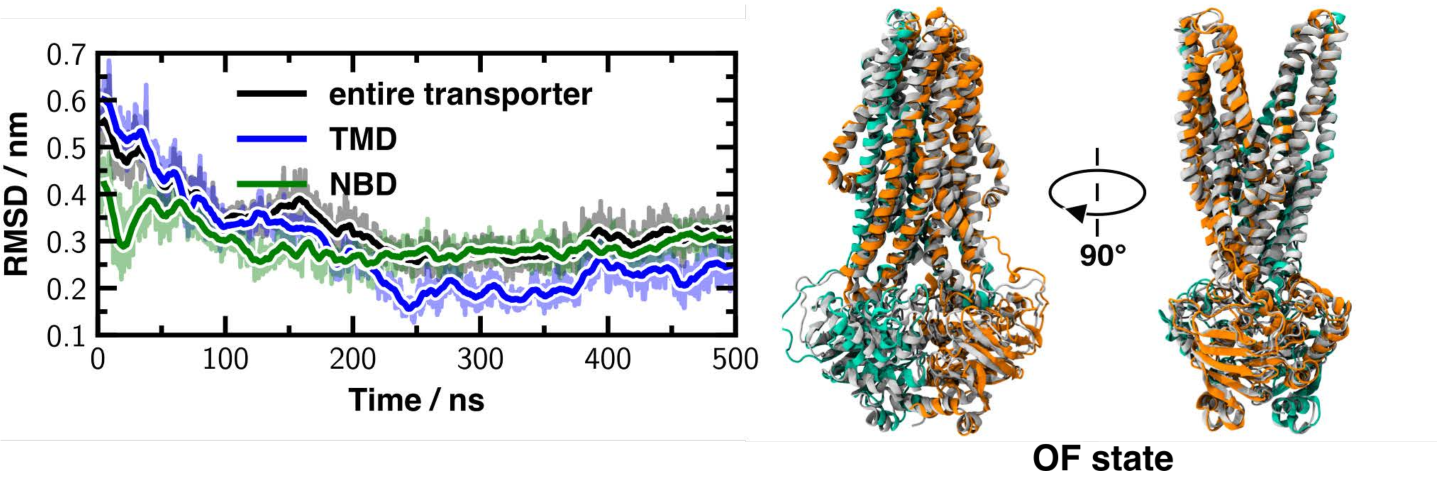

Supplementary Figure 5: Comparison of outward-facing TM287/288 predicted by MD simulation to the crystal structure. Left panel: RMSD between C_a-atoms of the OF crystal structure and the coordinates of the MD simulation during a representative IF-OF transition. Right panel: Superimposition of the predicted structure after 500 ns of MD simulation (TM287: orange, TM288: cyan) with the crystal structure of outward-facing TM287/288 (grey, PDB ID: 6QUZ).

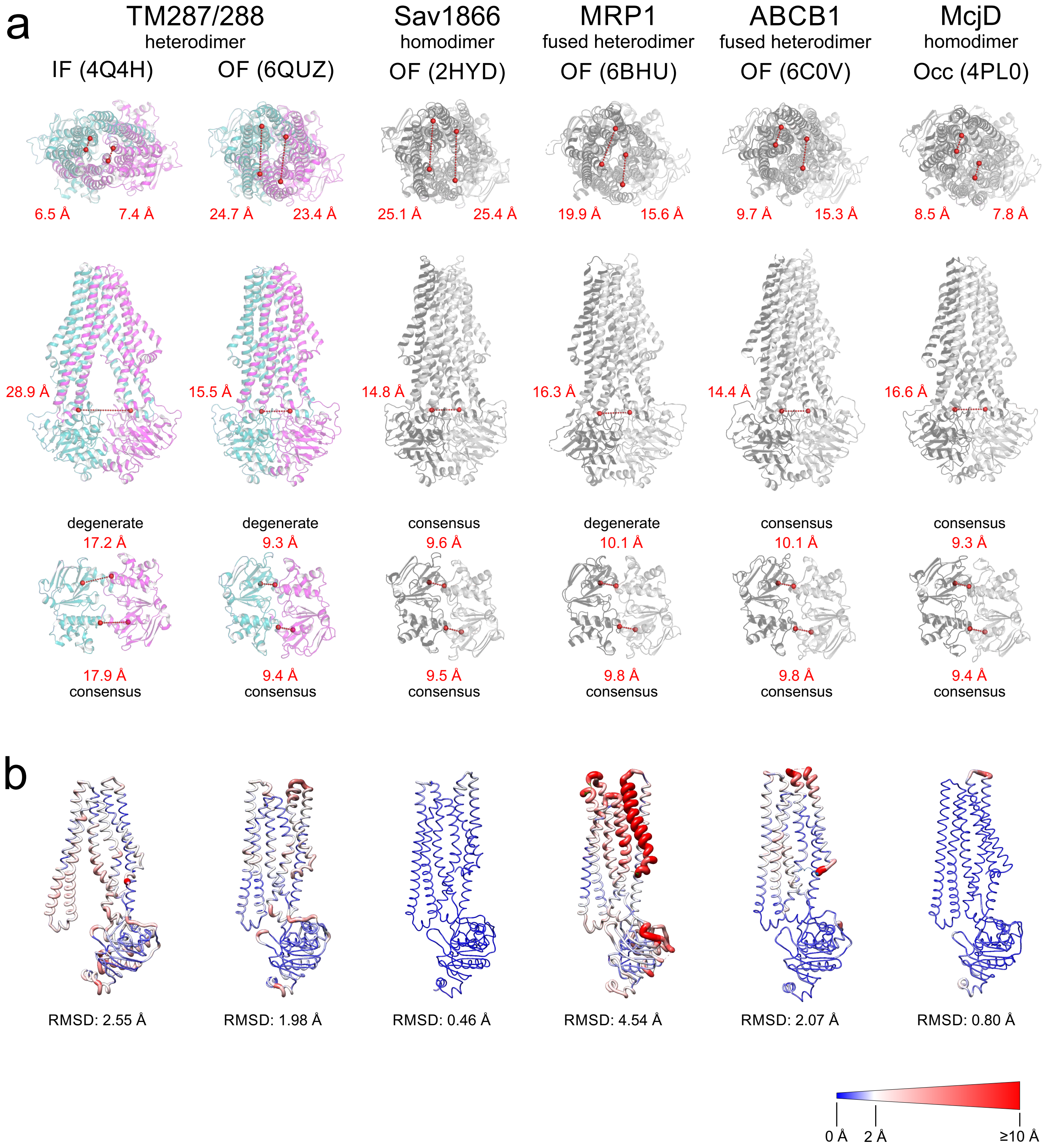

Supplementary Figure 6: Asymmetries of outward-facing and outward-occluded ABC exporters. High resolution structures of type I ABC exporters were analyzed with regard to asymmetries among the respective half-transporters. (**a**) Distances (C_α-C_α) were measured in the extracellular gate (top panel, residues corresponding to D41^{TM287}-E268^{TM287} and D65^{TM288}-T292^{TM288}, respectively), at the tetrahelix bundle (middle panel, residues corresponding to G201^{TM287}-G225^{TM288}), and the NBDs (bottom panel, distance between Walker A lysine and ABC signature serine in degenerate and consensus site, respectively). (**b**) Superimposition of the half-transporters shown in sausage representation. Greater thickness and red coloring correlate with larger structural deviations between equivalent positions.

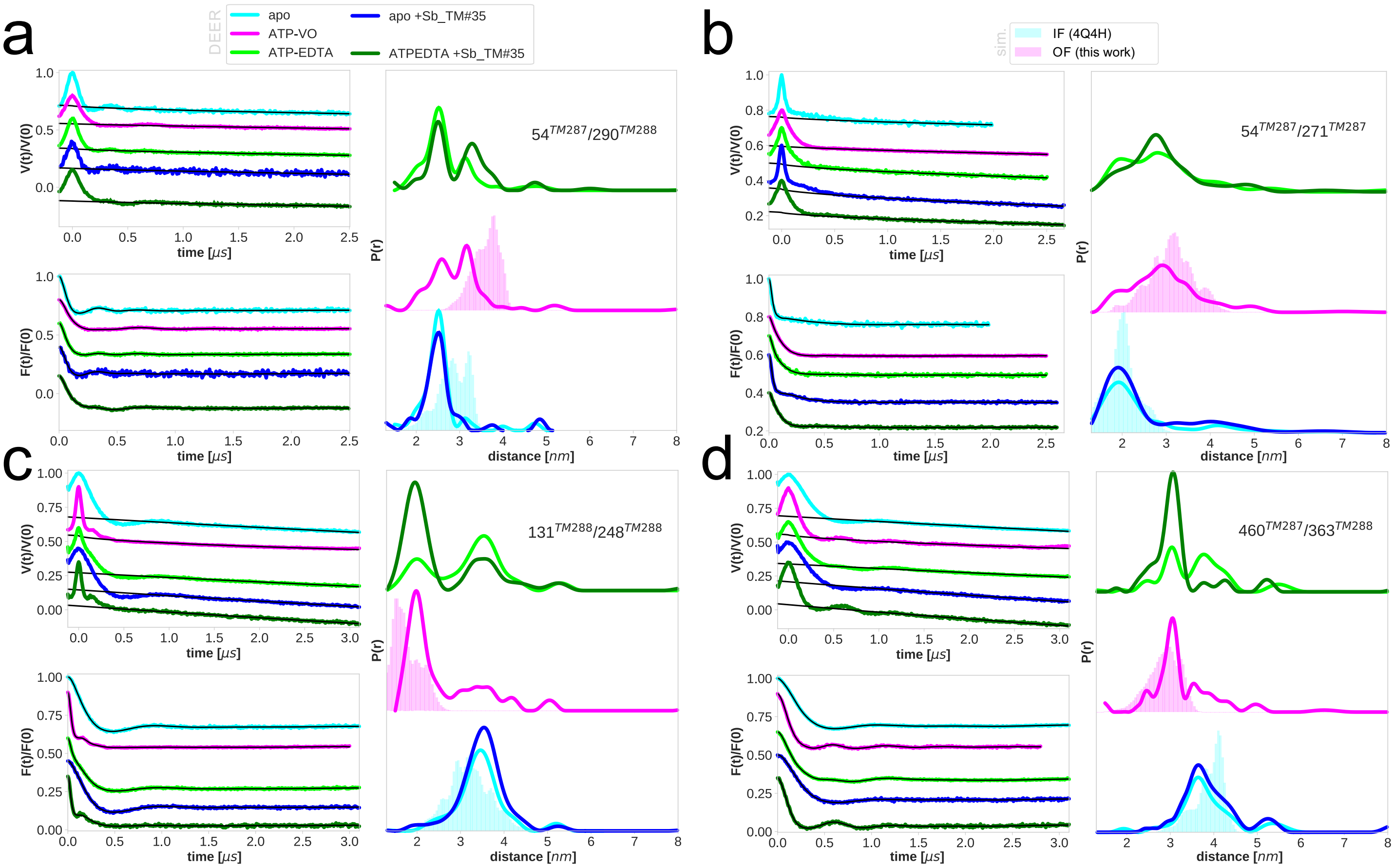

Supplementary Figure 7: Primary DEER traces of spin-labeled pairs in TM287/288 measured in the presence or absence of unlabeled Sb_TM#35. Each panel shows Q-band DEER traces [V(t)/V(0)] with the fitted background, background-corrected [F(t)/F(0)] traces with fitted distribution function and the corresponding area-normalized distance distributions calculated using DeerAnalysis2015. Measurements were conducted in the absence of nucleotide (apo) or in the presence of ATP and vanadate (ATP-VO) or ATP-EDTA. Distance distributions were simulated using the program MMM2015 based on the coordinates of the IF and OF structures of TM287/288. (**a** and **b**) Extracellular pairs $54^{TM287}/290^{TM288}$ and $54^{TM287}/271^{TM287}$. (**c**) Intracellular pair $131^{TM288}/248^{TM288}$. (**d**) NBD pair $460^{TM287}/363^{TM288}$.

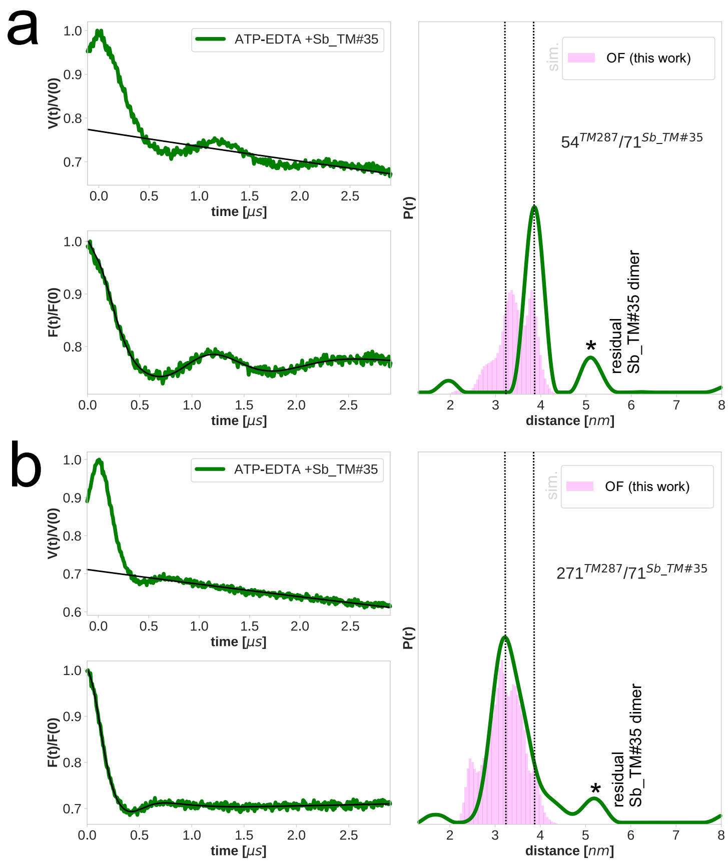

Supplementary Figure 8: Primary DEER traces of spin-labeled pairs in TM287/288 and Sb_TM#35. Each panel shows Q-band DEER traces [V(t)/V(0)] with the fitted background, background-corrected [F(t)/F(0)] traces with fitted distribution function and the corresponding area-normalized distance distributions calculated using DeerAnalysis2015. Measurements were conducted in the presence of ATP-EDTA. Distance distributions were simulated using the program MMM2015 based on the coordinates of the OF structure of TM287/288 in complex with Sb_TM#35. (**a**) DEER analysis of $71^{Sb\_TM\#35}$ and 54^{TM287} positioned on the same wing where Sb_TM#35 binds**.** (**b**) DEER analysis of $71^{Sb\_TM\#35}$ and 271^{TM287} positioned on the opposite wing. Asterisks denote the residual 5.2 nm peak confirmed to arise from sybody dimers in solution.

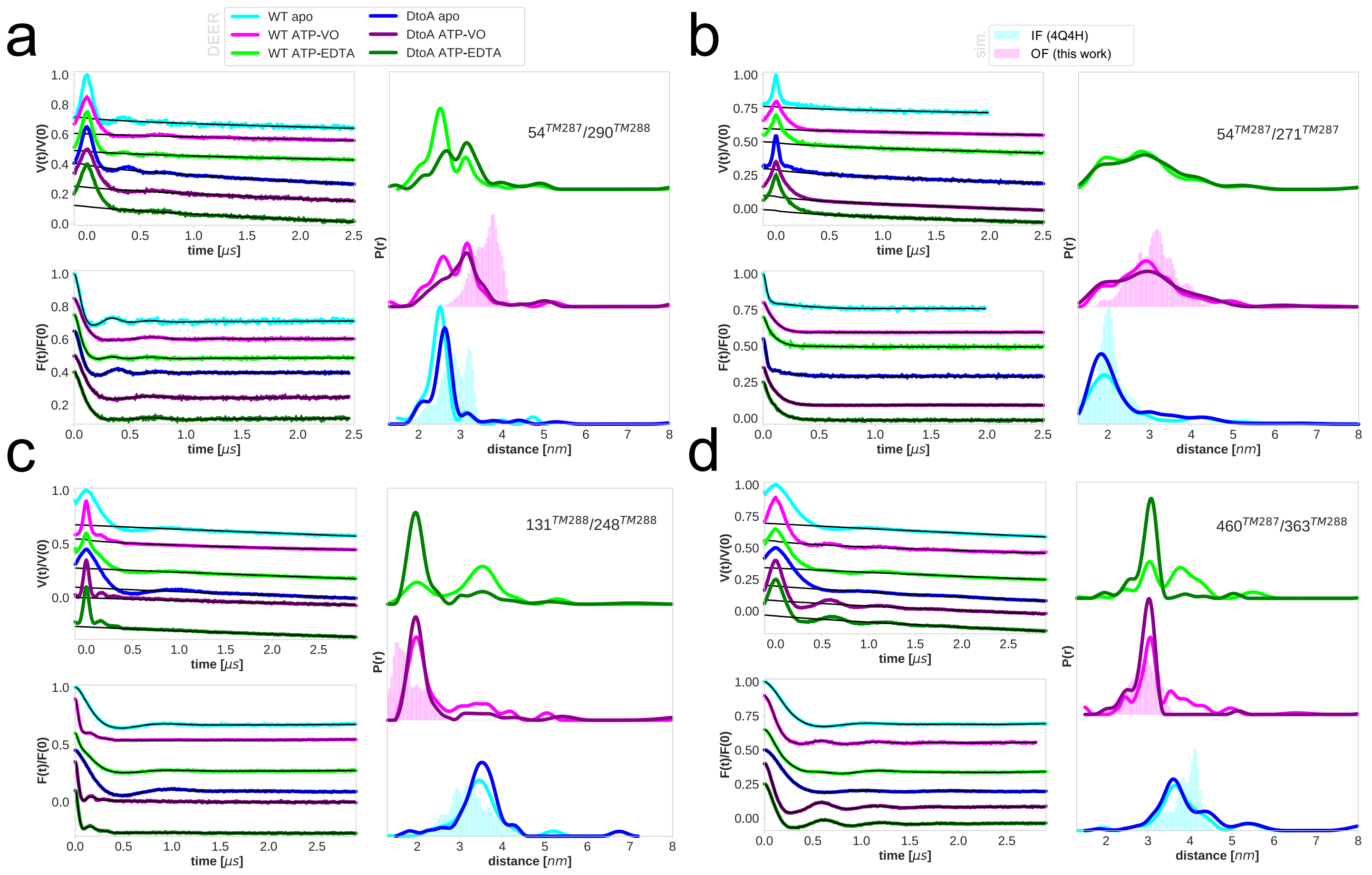

Supplementary Figure 9: Primary DEER traces of spin-labeled pairs of wildtype TM287/288 and TM287/288(2xDtoA). Each panel shows Q-band DEER traces [V(t)/V(0)] with the fitted background, background-corrected [F(t)/F(0)] traces with fitted distribution function and the corresponding area-normalized distance distributions calculated using DeerAnalysis2015. Measurements were conducted in the absence of nucleotide (apo) or in the presence of ATP and vanadate (ATP-VO) or ATP-EDTA. Distance distributions were simulated using the program MMM2015 based on the coordinates of the IF and OF structures of TM287/288. (**a** and **b**) Extracellular pairs $54^{TM287}/290^{TM288}$ and $54^{TM287}/271^{TM287}$. (**c**) Intracellular pair $131^{TM288}/248^{TM288}$. (**d**) NBD pair $460^{TM287}/363^{TM288}$.

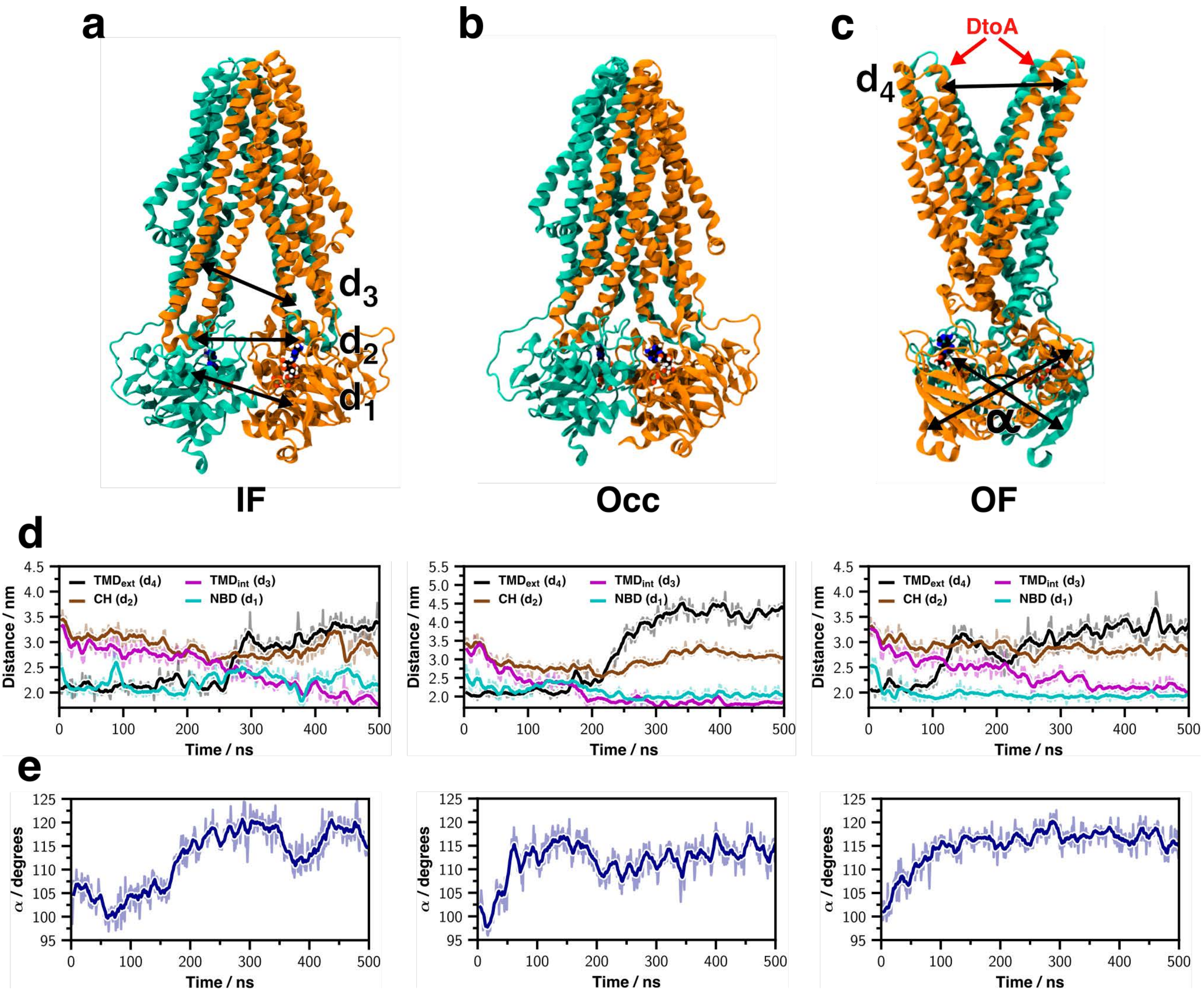

Supplementary Figure 10: IF-OF transition of TM287/288(2xDtoA) captured by MD simulations. (**a-c**) IF, Occ, and OF conformations sampled during MD simulation. C_a-C_a distances are measures for NBD dimerization (d_1: D460^{TM287}-S363^{TM288}), coupling helix motion (d_2: F127^{TM288}-T227^{TM288}), cytoplasmic gate closure (d_3: T131^{TM288}-S248^{TM288}) and extracellular gate opening (d_4: S50^{TM287}-S271^{TM287}). The arrows in (**c**) indicate the positions of mutated aspartates in the extracellular gate. (**d**) Distance time traces during three successful IF-to-OF conformational transitions. (**e**) Corresponding time traces of NBD-NBD twist angle, defined as the angle between the 2 vectors connecting C_a-atoms of L554^{TM287}-I452^{TM287} and L576^{TM288}-I474^{TM288}, respectively.

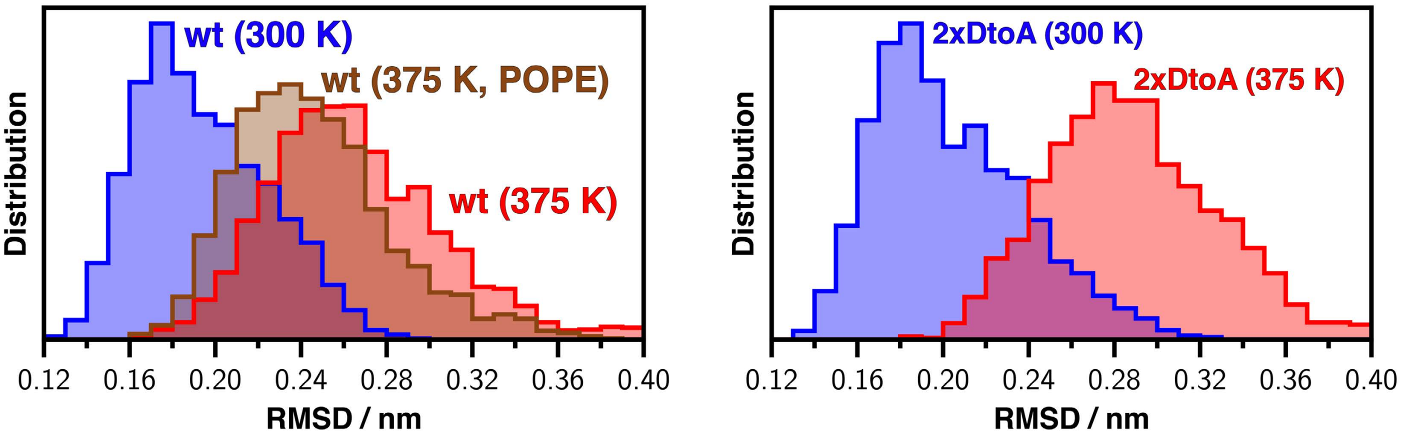

Supplementary Figure 11: Stability of OF crystal structure of TM287/288 during MD simulations. C_a-RMSD distribution from MD simulations of wildtype (left panel) and 2xDtoA mutant (right panel). Ten MD simulations (of 400 ns each) were carried out for both wildtype and 2xDtoA mutant at 300 K (blue) and at 375 K (red), respectively (i.e., 40 simulations in total). In addition, ten MD simulations (500 ns each) were carried out for the wildtype in a POPE (instead of POPC) bilayer at 375 K.

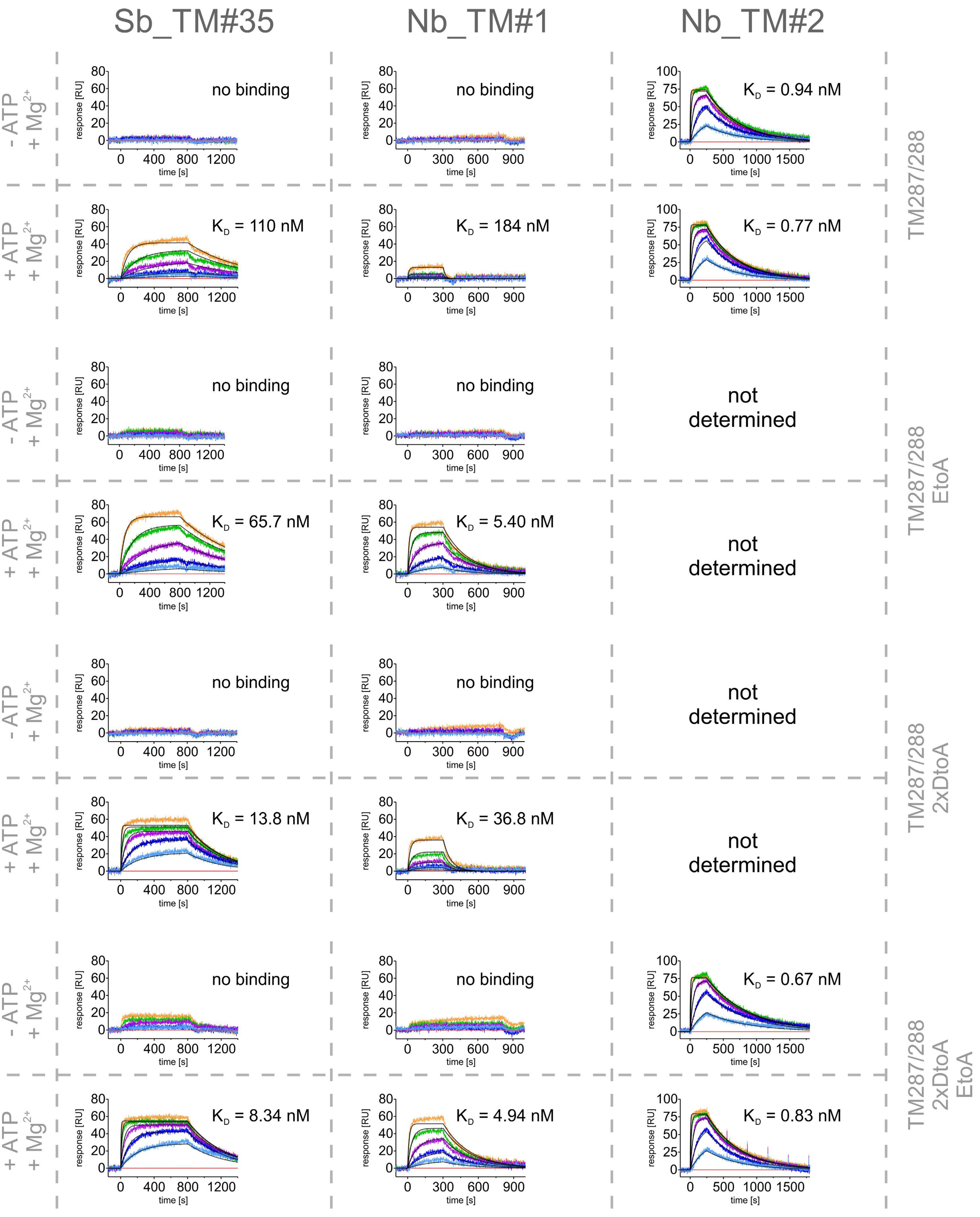

Supplementary Figure 12: SPR analysis of single domain antibodies. TM287/288 and the respective mutants were immobilized on the SPR sensor chip and Sb_TM#35, Nb_TM#1 and Nb_TM#2 served as analytes. Binding experiments were conducted in an Mg^{2+}-containing buffer in the presence or absence of ATP. Injected concentrations: Sb_TM#35: 0, 9, 27, 81, 243, 729 nM; Nb_TM#1: 0, 1, 3, 9, 27, 81 nM; Nb_TM#2: 0, 0.9, 2.7, 8.1, 24.3, 72.9 nM. Kinetic analyses are shown in Supplementary Table 2.

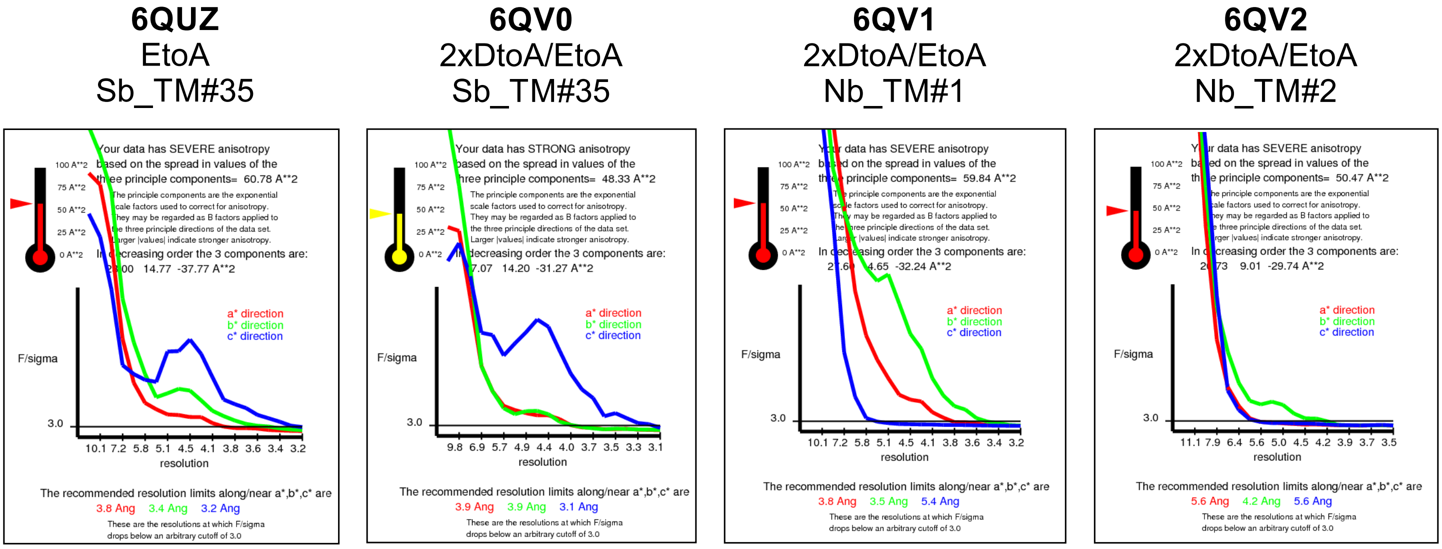

Supplementary Figure 13: Crystallographic diffraction data truncation using the diffraction anisotropy server. Truncations were carried out using standard settings (F/sigma ≥ 3.0).
