# Supplementary material for: The extracellular gate shapes the energy profile of an ABC exporter
Source: Nat Commun. 2019 May 21;10:2260. doi: 10.1038/s41467-019-09892-6 (PMC6529423; doi:10.1038/s41467-019-09892-6)
Supplement: Supplementary file 3 — Reporting Summary [file 41467_2019_9892_MOESM3_ESM.pdf]

nature research

Corresponding author(s): Enrica Bordignon, Markus A. Seeger

Reporting Summary

Nature Research wishes to improve the reproducibility of the work that we publish. This form provides structure for consistency and transparency in reporting. For further information on Nature Research policies, see Authors & Referees and the Editorial Policy Checklist.

Statistical parameters

When statistical analyses are reported, confirm that the following items are present in the relevant location (e.g. figure legend, table legend, main text, or Methods section).

n/a	Confirmed	
☒	☐	The exact sample size (n) for each experimental group/condition, given as a discrete number and unit of measurement
☐	☒	An indication of whether measurements were taken from distinct samples or whether the same sample was measured repeatedly
☒	☐	The statistical test(s) used AND whether they are one- or two-sided<br>*Only common tests should be described solely by name; describe more complex techniques in the Methods section.*
☒	☐	A description of all covariates tested
☒	☐	A description of any assumptions or corrections, such as tests of normality and adjustment for multiple comparisons
☐	☒	A full description of the statistics including central tendency (e.g. means) or other basic estimates (e.g. regression coefficient) AND variation (e.g. standard deviation) or associated estimates of uncertainty (e.g. confidence intervals)
☒	☐	For null hypothesis testing, the test statistic (e.g. F, t, r) with confidence intervals, effect sizes, degrees of freedom and P value noted<br>*Give P values as exact values whenever suitable.*
☒	☐	For Bayesian analysis, information on the choice of priors and Markov chain Monte Carlo settings
☒	☐	For hierarchical and complex designs, identification of the appropriate level for tests and full reporting of outcomes
☒	☐	Estimates of effect sizes (e.g. Cohen's d, Pearson's r), indicating how they were calculated
☐	☒	Clearly defined error bars<br>*State explicitly what error bars represent (e.g. SD, SE, CI)*

Our web collection on statistics for biologists may be useful.

Software and code

Policy information about availability of computer code

Data collection	*Provide a description of all commercial, open source and custom code used to collect the data in this study, specifying the version used OR state that no software was used.*
Data analysis	Excel, SigmaPlot, GraphPad Prism 5.04, BioRad Proteon Analysis Software, SB Grid (XDS, Phenix, ccp4, Buster), GROMACS version 5.1, DeerAnalysis2015, MMM2015, Pymol, Chimera

For manuscripts utilizing custom algorithms or software that are central to the research but not yet described in published literature, software must be made available to editors/reviewers upon request. We strongly encourage code deposition in a community repository (e.g. GitHub). See the Nature Research guidelines for submitting code & software for further information.

Data

Policy information about availability of data

All manuscripts must include a data availability statement. This statement should provide the following information, where applicable:

- Accession codes, unique identifiers, or web links for publicly available datasets
- A list of figures that have associated raw data
- A description of any restrictions on data availability

The structures have been deposited in the PDB under accession numbers: 6QUZ (Sb_TM#35, ATPγS-bound), 6QV0 (Sb_TM#35, ATP-bound), 6QV1 (Nb_TM#1) and

6QV2 (Nb_TM#2).
Sybody Sb_TM#35 and nanobodies Nb_TM#1 and Nb_TM#2 as well as all other genetic constructs will be made available upon reasonable request to the corresponding authors.

Field-specific reporting

Please select the best fit for your research. If you are not sure, read the appropriate sections before making your selection.

☒ Life sciences ☐ Behavioural & social sciences ☐ Ecological, evolutionary & environmental sciences

For a reference copy of the document with all sections, see nature.com/authors/policies/ReportingSummary-flat.pdf

Life sciences study design

All studies must disclose on these points even when the disclosure is negative.

Sample size	Sample size estimation was not relevant for this study, as it does not report on a statistical evaluation of effects between two or more groups.
Data exclusions	No data were excluded from the analysis
Replication	ATPase activity measurements were carried out with three technical replicates for each data point and these data were used to calculate mean and standard deviations (error bars in Fig. 2b; Fig. 4c,d,g; Fig. S2b). In case ATPase data shown in Fig. S2a, technical duplicates were measured.<br><br>SPR assays were performed with one replicate for each sybody/nanobody concentration. The SPR traces shown in Fig. 1d were repeated at least twice as biological replicates.<br><br>DEER assays (Fig. 2c,d; Fig. 4f; Fig S7-S9) were performed at least twice with biological replicates (different protein batches for TM287/288 and Sb_TM#35).<br><br>Transport experiments with EfrEF were repeated with two independent cell batches and with two technical replicates for each batch (Fig. 4e).<br><br>The conformational probing experiment (Fig. 5) was repeated twice with two different sybody/nanobody samples.<br><br>MD simulations for the IF-to-OF conversion (Fig. S10) were performed 20 times.<br>The MD simulation looking at the stability of OF TM287/288 and TM287/288_2xDtoA was performed 10 times each at 300 K and 275 K (Fig. S11).
Randomization	No randomization was performed.
Blinding	Experiments were not blinded.

Reporting for specific materials, systems and methods

Materials & experimental systems

n/a	Involved in the study
☐	☒ Unique biological materials
☐	☒ Antibodies
☒	☐ Eukaryotic cell lines
☒	☐ Palaeontology
☐	☒ Animals and other organisms
☒	☐ Human research participants

Methods

n/a	Involved in the study
☒	☐ ChIP-seq
☒	☐ Flow cytometry
☒	☐ MRI-based neuroimaging

Unique biological materials

Policy information about availability of materials

Obtaining unique materials	Sybody Sb_TM#35 and nanobodies Nb_TM#1 and Nb_TM#2 recognizing TM287/288 were generated as part of this manuscript.

Obtaining unique materials	These will be made accessible to interested labs upon request.

Antibodies

Antibodies used	mouse monoclonal anti myc, Sigma #M4439 ELISA
Validation	The antibody was tested for specificity in ELISA with purified target proteins.

Animals and other organisms

Policy information about studies involving animals; ARRIVE guidelines recommended for reporting animal research

Laboratory animals	A female 2 year old alpaca (Vicugna pacos) called Oxa was used for immunization with TM287/288.
Wild animals	*Provide details on animals observed in or captured in the field; report species, sex and age where possible. Describe how animals were caught and transported and what happened to captive animals after the study (if killed, explain why and describe method; if released, say where and when) OR state that the study did not involve wild animals.*
Field-collected samples	*For laboratory work with field-collected samples, describe all relevant parameters such as housing, maintenance, temperature, photoperiod and end-of-experiment protocol OR state that the study did not involve samples collected from the field.*
